# Multiphoton lithography with protein photoresists

**DOI:** 10.1016/j.mtbio.2024.100994

**Published:** 2024-02-10

**Authors:** Dmitry Sivun, Eljesa Murtezi, Tina Karimian, Kurt Hurab, Maryam Marefat, Elena Klimareva, Christoph Naderer, Boris Buchroithner, Thomas A. Klar, Georgii Gvindzhiliia, Andreas Horner, Jaroslaw Jacak

**Affiliations:** a Department of Medical Engineering, University of Applied Sciences Upper Austria, Garnisonstraße 21, 4020, Linz, Austria; b Institute of Applied Physics, Johannes Kepler University Linz, Altenberger Straße 69, 4040, Linz, Austria; c Institute of Biophysics, Johannes Kepler University Linz, Gruberstraße 40, 4020, Linz, Austria; d AUVA Research Center, Ludwig Boltzmann Institute for Experimental and Clinical Traumatology, Donaueschingenstraße 13, 1200 Vienna, Austria

**Keywords:** Multiphoton lithography, Protein printing, Functional photoresist, Tissue scaffolds, Extracellular vesicle

## Abstract

Recently, 2D/3D direct laser writing has attracted increased attention due to its broad applications ranging from biomedical engineering to aerospace. 3D nanolithography of water-soluble protein-based scaffolds have been envisioned to provide a variety of tunable properties. In this paper, we present a functional protein-based photoresist with tunable mechanical properties that is suitable for multiphoton lithography (MPL). Through the use of methacrylated streptavidin or methacrylated bovine serum albumin in combination with polyethylene glycol diacrylate or methacrylated hyaluronic acid as crosslinkers and a vitamin-based photoinitiator, we were able to write two- and three-dimensional structures as small as 200 nm/600 nm lateral/axial features, respectively. We also demonstrated that Young's modulus can be tuned by the photoresist composition, and we were able to achieve values as low as 40 kPa. Furthermore, we showed that Young's modulus can be recovered after drying and rehydration (i.e. shelf time determination). The retained biological functionality of the streptavidin scaffolds was demonstrated using fluorescently labelled biotins. Using single-molecule fluorescence microscopy, we estimated the density of streptavidin in the written features (1.8 ± 0.2 × 10^5^ streptavidins per 1.00 ± 0.05 μm³ of feature volume). Finally, we showed applicability of our 2D scaffold as a support for a fluorescence absorbance immuno-assay (FLISA), and as a delivery platform of extracellular vesicles to HeLa cells.

## Graphical abstract

**Figure:**
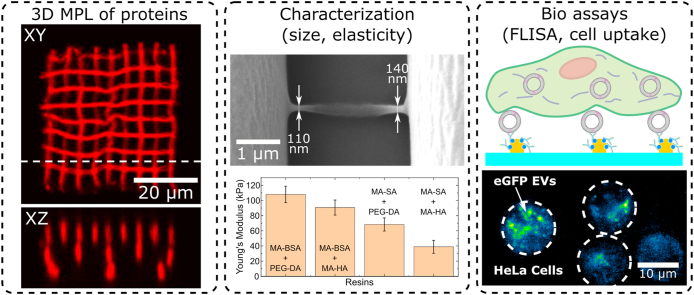

## 1 Introduction

Over the last decade, additive manufacturing has rapidly developed, and with 2D/3D direct laser writing (DLW) gained importance due to its broad applicability ranging from biomedical engineering to aerospace [1]. The versatility of DLW originates mainly from the variable wavelength of the excitation source and the diversity of the processed materials [1,2]. Thus, structuring from millimeter down to the sub-micrometer scale is possible using 2D/3D DLW [2]. Multiphoton lithography (MPL), optionally combined with stimulated emission depletion (STED), is the only current method providing sufficient spatial resolution for 3D structuring in the nanometer range [3,4]. However, nanometer scale 3D MPL has been realized mostly with a limited number of synthetic monomers functionalized via reactive backbone modifications [[5], [6], [7], [8]]. 3D nano-structuring of functional biocompatible polymers still represents a major challenge [9]. More recently, hydrogel-based photoresists made of natural biopolymers have garnered significant attention because of their unique properties, that provide for their use as promising materials for numerous applications in tissue engineering, drug delivery, and cosmetics [10]. Hydrogel-based microstructures exhibit dynamic properties such as actuation and shape-shifting capabilities under different stimuli including pH, solvent, light, or temperature changes [[11], [12], [13], [14], [15]]. Structuring of hydrogels in the micrometer regime has been yet demonstrated only in 2D/2.5D [[11], [12], [13]], True 3D scaffolds were only demonstrated in the sub-millimeter size range [16] (except scaffolds made of gelatin, which is non-functional denatured protein). Downscaling hydrogel-based structures to the sub-micrometer range brings several advantages for controlling cellular behavior [17], and for a more precise tuning of the mechanical or optical properties of the materials [18]. So far, only a few natural polymers have been used, mostly for 2D diffraction limited printing [[19], [20], [21], [22], [23], [24]]. 3D MPL patterning of biomaterials has been mostly realized with synthetic biocompatible molecules [25], amino acids [26], or semi-synthetic monomers [27]. Protein-based 3D scaffolds were created either via modified proteins [16] or direct cross-linking of the proteins [28]. Modified proteins (e.g. silk methacrylate (SilMA)) or bovine serum albumin methacrylate (BSAMA) have been structured [16], but in sizes of hundreds of micrometers.

To date, direct cross-linking (i.e. photoinitiator-free) has been realized with bovine serum albumin (BSA) [28] and applied for cell cultivation [13,[29], [30], [31]]. The mechanism behind the direct cross-linking of the proteins as well as indirect linking of protein-polymer hybrid structures has been studied for several proteins. It has been demonstrated that the laser power required for MPL clearly depends on protein composition and can affect the protein structure [20,32]. Cross-reactivity between proteins is typically introduced by amino acids such as tryptophan, tyrosine or cysteine, and is mediated by singlet oxygens [33,34]. In addition to cross-reactivity, there are also π-π* transitions of aromatic residues such as tyrosine and phenylalanine, which lead to absorption at 260 nm which damages them [32]. This has been also demonstrated by the analysis of light damage and photobleaching in cells [35,36]. The thermal cross-linking efficiency/density thus results from the use of reactive sites of the protein molecules. There is also the possibility of proteins being entrapped in a polymeric 3D hydrogel. In the case of direct protein polymerization, the process is rather slow and requires non physiological solvents, which might influence the protein structure [27,28]. Consequently, the mechanical properties, such as Young's modulus, correlate with the cross-linking rate of the protein-based materials as well as with the amino acid sequence of the proteins [27,37]. Therefore, further research on 3D submicron-lithography of protein-based structures with tunable properties (e.g. Young's modulus, surface reactivity) out of water-soluble photoresist is highly relevant [10,12,25,38,39].

In this contribution, we present a functional protein-based photoresist which allows for multiphoton lithography (MPL) and its applications. By using methacrylate-modified proteins and a vitamin-based photoinitiator, we were able to write two- and three-dimensional structures with Young's moduli and lateral/axial feature sizes as small as 40 kPa and 200 nm/600 nm, respectively. The photoresists consist of methacrylated protein (streptavidin or bovine serum albumin, both methacrylated in house as none of them is commercially available). Polyethylene-glycol diacrylate or methacrylated hyaluronic acid is used as cross-linker for additional stiffness modulation. For the first time, a functional protein such as streptavidin was employed in the structuring of 2D/3D scaffolds. The functionality and density of streptavidin inside the printed structures was demonstrated via binding of fluorescently labelled biotin and quantitative single-molecule fluorescence microscopy analysis. The protein-based structures withstood several dehydration-rehydration cycles. We show that mechanical properties as well as the functionality of protein-based photoresists can be tuned by the photoresist composition and can be restored after drying by rehydration, which creates an extended shelf life. 10.13039/100014337Furthermore, we demonstrate the applicability of streptavidin-based structures as a support for a fluorescence absorbance immuno-assay (FLISA) to detect EV membrane proteins. Lastly, we illustrate that similar streptavidin-based structures can also be used to study the selective uptake of immobilized extracellular vesicles into cells.

## 2 Results and discussion

### 2.1 Fabrication and characterization of protein-based structures

**Fig. 1:**
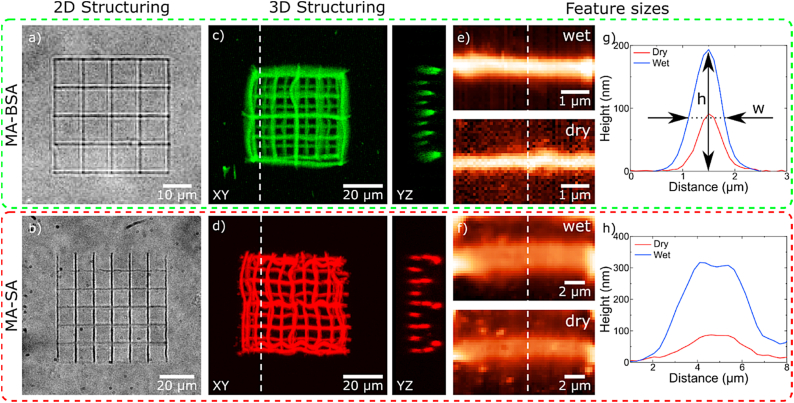
**2D/3D structuring of protein-based photoresists.** a) and b) show bright field images of MA-BSA and MA-SA 2D grids, respectively. c) and d) depict confocal fluorescence images of 3D grids in aqueous environment fabricated out of MA-BSA and MA-SA. The panels on the right-side display YZ-plane cross-sections (along the white dashed lines), where three different grid constants are visible. e) and f) atomic force microscopy (AFM) images of MA-BSA and MA-SA lines in aqueous environment (upper panel) and dry state, stored at 25 °C for 5 days, (lower panel) showing achievable lateral and axial dimensions, respectively. g) the cross-section of MA-BSA lines in wet and dry conditions (white dashed line in e). h) the cross-section of MA-SA lines in wet and dry conditions (white dashed line in f). The lateral and axial dimensions of printed lines are represented by height (h) and width at FWHM (w), respectively.

MPL structuring of our new protein-based water soluble photoresists, consisting of a methacrylated protein (30%, bovine serum albumin (MA-BSA) or streptavidin (MA-SA)), a cross-linker (5%, polyethylene glycol diacrylate (PEG-DA) or methacrylated hyaluronic acid (MA-HA)), and a photoinitiator (0.25%, riboflavin 5′-monophosphate sodium salt (vitamin B2) or alternatively Lithium phenyl-2,4,6-trimethylbenzoylphosphinate (LAP)), was performed via radical polymerization using a 515 nm fs pulsed laser. 2D/3D structures were printed to depict the 2D/3D MPL writing capabilities of the presented protein-based photoresists. Fig. 1a and b shows bright-field images of 2D grids (grid constant 10 μm), fabricated out of MA-BSA and MA-SA (with PEG-DA as cross-linker), respectively. Fig. 1c and d shows confocal fluorescence images of the woodpile-like structures, consisting of three grids with grid constants of 20, 10, and 5 μm, shifted in the z-direction with respect to each other by 2 μm. Thus, the smallest grid was elevated ∼12 μm above the glass surface. Each individual bar in this structure was fabricated by multiple illuminations with a small overlap of the excitation volume (500 nm distance between the PSF centers). The side panels in Fig. 1c and d displays YZ cross-sections of the free hanging lines. The woodpile-like structures are highly flexible, which leads to shape irregularities, nevertheless they show stability of at least two weeks in water.

In order to investigate minimal MPL written feature sizes, 3D suspended lines, similar to the ones used by Buchroithner et al. [18], were produced (see SI). Three parallel supporting bars (100 μm long and 5 μm tall) made of OrmoComp® were fabricated on a glass cover slip. The lines were spaced by 50 and 2 μm. Next, perpendicular to the OrmoComp® supporting bars, protein-based freely suspended lines (FIG. S1) were written (60 μm long and 3 μm above the glass surface). Each sample contained 15 lines written with varying peak intensities. During the development (washing), the protein-based lines tilted (rotated) by 90° and lay flat on the glass surface. This arrangement simplified the quantification of the line dimensions and Young's modulus in a reproducible way [18]. Atomic force microscopy (AFM) was applied to simultaneously analyze the lateral and axial dimensions as well as Young's modulus of the tipped protein-based lines. Herein, the height (h) and the width (w, at FWHM) of the measured lines represents their lateral and axial dimensions, respectively (see Fig. 1g and FIG. S1c,d for a detailed explanation of measurement configuration). Fig. 1e and f shows AFM images of the smallest measured lines. For MA-BSA (wet state), the lateral and axial dimensions were 205 ± 26 nm and 613 ± 54 nm, respectively. In the case of MA-SA (wet state), lateral 349 ± 14 nm and axial 2723 ± 271 nm were measured. The dimensions of the same lines in dry state are presented in Table S1. The dimensions (lateral and axial) were confirmed via scanning electron microscopy (SEM). FIG. S1b shows an example of axial and lateral feature sizes measured for MA-BSA lines (∼10 nm additional metal coating). The theoretical diameter (1/e^2^) of the two-photon excitation volume (515 nm excitation wavelength and objective: 63×, NA = 1.2) is ∼275 nm in lateral and ∼835 nm and axial direction, respectively [40]. While experimentally achieved dimensions for MA-BSA lines are in agreement with theoretical values, the lines made of MA-SA exhibit larger dimensions, specifically in axial direction. In one aspect, the larger dimensions (compared to the theoretical values) of the MA-SA lines can be attributed to the remaining gel phase of the regrown, not fully crosslinked polymer material on their surface, as they were not fully washed during the development of the structures. However, this effect was not observed for the MA-BSA lines, even though the washing procedure was identical. In another aspect, the non-proportional increase in lateral and axial dimensions could be explained by the adhesion of the proteins to the glass substrate, which might lead to a "flattening" of the inclined lines, resulting in a decrease in height (lateral) and an increase in width (axial).

**Fig. 2:**
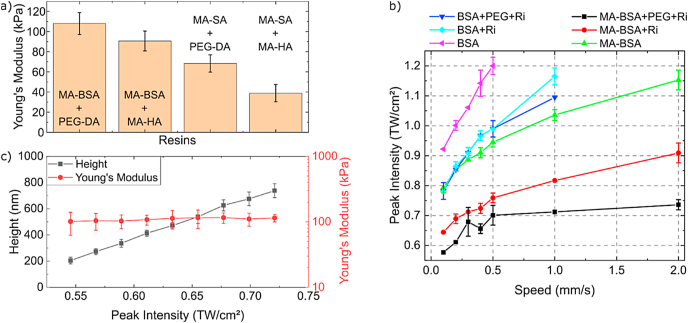
**Mechanical properties of the protein-based resins and writing threshold.** a) Young's Modulus of 4 different resin compositions in wet conditions (n_total_ = 45; 15 points on one line, 3 technical replicas; (4 technical replicas for MA-SA + PEG-DA compositions)) b) writing threshold as a function of writing peak intensity and writing speed for 6 different resin compositions. c) height and Young's moduli of MA-BSA lines as function of the writing peak intensity.

To access Young's modulus, we carried out nanoindentation experiments. Specifically, force-distance curves were measured at each pixel and fitted with the Hertz model [41]. Point-probe FMR cantilevers were employed as indenters and their exact spring constant and sensitivity were determined before each experiment. Fig. 2a shows Young's Moduli for all tested resin compositions in an aqueous environment. In general, all tested resins show Young's Moduli in the kPa range. However, compositions containing MA-SA showed approximately 2× lower Young's modulus (∼40 kPa) compared to the composition based on BSA. We attribute this to the reduced cross-linking density of the MA-SA composition due to fewer methacrylated groups on each protein. Streptavidin contains 5 NH_2_ groups per subunit (20 in total) [42] whereas bovine serum albumin has ∼30–35 sterically accessible amino groups [43]. It is important to note that we do not functionalize all accessible amino groups as this would inevitably lead to protein unfolding and loss of bio functionality [32]. According to trinitrobenzene sulfonic acid (TNBS) assay (details in SI), we achieved a functionalization efficiency of ∼33% for BSA and ∼14% for SA, which corresponds to ∼10 and ∼3 methacrylated groups per BSA and SA molecule, respectively. Thus, MA-SA had 3× fewer amino groups contributing to the cross-linking compared to MA-BSA. This could also be a reason for the poor washing behavior previously mentioned and the associated larger dimensions of the MA-SA structures.

To avoid the unwanted direct cross-linking of the protein's residues, we analyzed the MPL writing threshold for six different compositions: three with methacrylate modified proteins (MA-BSA, MA-BSA + Riboflavin, and MA-BSA + PEG-DA + Riboflavin) and three non-modified proteins (BSA, BSA + Riboflavin, and BSA + PEG-DA + Riboflavin). Fig. 2b provides information on the writing thresholds for modified and unmodified protein structures as a function of writing speed and writing peak intensity. Lower writing thresholds were observed for methacrylate modified proteins indicating that the cross-linking occurs mainly by free radical chain polymerization. Although thermally induced processes (direct π - π* transitions of aromatic residues such as tyrosine and phenylalanine) that lead to direct protein cross-linkage cannot be entirely excluded, they are less probable due to almost 2× higher writing threshold values of non-methacrylated proteins compared to methacrylated proteins [44,45]. To prove that predominantly methacrylated groups are crosslinked, we measured the size and Young's moduli of MA-BSA tipped lines as a function of writing peak intensity (Fig. 2c). A linear correlation of the lateral feature size (from 200 to 750 nm) with the writing peak intensity was observed. In contrast, the Young's modulus data showed independence of peak intensity and remained constant (∼100 kPa). Similar behavior was also observed for MA-SA structures. Our results indicate that the used peak intensities were sufficient to primarily crosslink methacrylate groups. The elasticity of the suspended lines was also shown by stretching individual, anchored lines with an AFM tip (see movie S1). The stretching experiment yielded a 2.5-fold increase of the line length and was hindered by the detachment of the line from the OrmoComp® supporting bars.

**Fig. 3:**
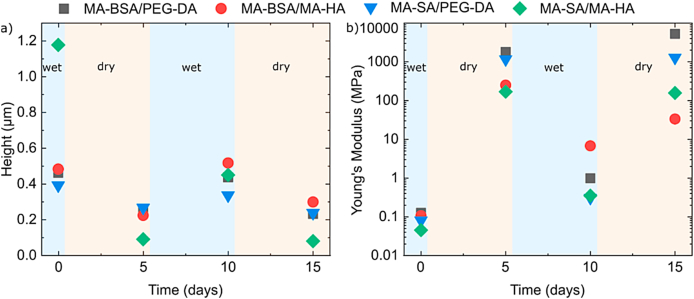
**Mechanical properties of the protein-based resins.** a) and b) shows the height and Young's moduli of lines made of four different protein-based resists as a function of the environmental conditions. For MA-SA/PEG-DA in the second cycle only one technical replica was used.

Next, we quantified the response of the materials to environmental changes (drying) which is relevant for long-term storage. To achieves this, environment-dependent changes in the Young's moduli were investigated, as well as the ability of the material to recover its original mechanical properties. Fig. 3 shows changes of the height and the Young's moduli of individual nanostructures (lines) for four protein-based resists during multiple wet/dry cycles. The first measurements were performed under wet conditions directly after fabrication. Subsequently, the samples were dehydrated (removal of water and storage at 25 °C for 5 days) and measured under dry conditions. The entire environmental change cycle was repeated two times.

Generally, Young's moduli and sizes behaved comparably for all investigated formulations. Dehydration always led to a reduction of the feature size and an increase of the Young's modulus, whereas subsequent hydration almost fully restores initial line parameters. Formulations with MA-HA demonstrated a larger variation in height between wet and dry states, with changes of 53% and 92% for MA-BSA/MA-HA and MA-SA/MA-HA, respectively. While formulations containing PEG-DA exhibit a smaller average modulation of about 38%. Additionally, we observed an average difference of ∼8% between the wet states in the 1st and 2nd cycles for all formulations, except for MA-SA/MA-HA, where this change was as much as 60%. In contrast, the difference in Young's Modulus between the two wet states was more significant. We observed an average ∼6-fold increase of YM for all formulations, except for MA-BSA/MA-HA, where the change was 68 times. Overall, these differences between wet states can most likely be attributed to the washing out of only mechanically trapped proteins during the first washing cycle, which is indirectly confirmed by the reduction of the line's sizes in the second wet cycles. It is worth mentioning that the amplitude of Young's modulus changes between wet and dry states was also significantly larger compared to the modulation in height. Formulations contained MA-HA and PEG-DA showed YM increase in average of ∼3000 and 16000 times respectively. In absolute values, the Young's modulus of photoresist formulations containing PEG-DA was nearly an order of magnitude higher in the dry state (in all cycles) compared to formulations including MA-HA. This behavior is explained mainly by the shorter length of the PEG-DA cross-linker, since it has been shown previously that elasticity depends on chain length [[46], [47], [48], [49], [50]]. The hydration layer formed around PEG-DA and HA is another mechanism that can affect elasticity [50,51]. Due to their different water solubilities, each of the molecules may have a different hydration layer. The same argument holds true for cross-linked/captured proteins in the photoresist [52,53]. Published data on BSA show that the volume of a non-hydrated (dry) BSA molecule was determined [52,54] as *v*_*dry*_ = 81 nm³ which is more than 2× smaller compared to its hydrated state (*v*_*hydrated*_ = 192 nm³) [55,56]. When taking all these factors into account, it was not surprising that the MA-SA/MA-HA formulation showed the smallest Young's modulus (∼40 kPa) among all formulations. We also observed that the photoresists containing MA-HA (regardless of the protein) exhibited a greater decrease in structure height (compared to formulations with PEG-DA) during drying (up to ∼12-fold), which indicates a higher hydration of HA. Variations in osmolarity, ionic strength, solvents, etc. will affect the feature size and mechanical properties of the protein polymers. Such a contribution could be of interest in tissue engineering applications (mimicking of ECM properties), aging, and senescence of ECM.

**Fig. 4:**
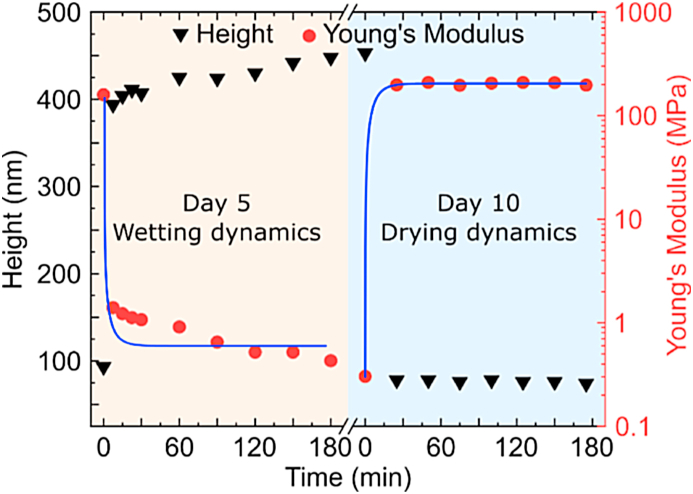
**Dynamics of environmentally induced changes in the size and young's modulus of protein-based resin.** The blue solid lines of the fit show a general trend of the curve. The experiments were conducted under standard laboratory conditions (24 °C, 40% humidity).

Fig. 4 depicts the temporal analysis of height and Young's moduli of MA-SA lines during wetting and drying. Our results show that wetting and drying are very fast processes, happening within a few minutes (even below our time resolution, which is limited by AFM image acquisition time). However, wetting and drying dynamics are not identical. Despite the fast alteration at the beginning of wetting, it takes several hours to attain constant values of height and Young's modulus. Conversely, during drying, these values become immediately constant. We attribute such fast dynamics to the sub-μm size of the structures.

MA-SA expresses the intrinsic functionality of streptavidin in the scaffold. Using fluorescence microscopy and fluorescently labelled biotin (ATTO655-biotin), we showed that the functionality of the protein remains after methacrylation and 3D MPL printing (Fig. 1d). FIG. S2a-c shows bright field and fluorescence images of a 2D MA-SA grid before and after incubation with ATTO655-biotin, respectively. Incubating the structure with ATTO655-biotin increased its fluorescence by 4 orders of magnitude compared to the background fluorescence. The measured average intensity before incubation was 118 ± 15 counts/pixel and 1.89 ± 0.37 × 10^6^ counts/pixel afterwards (after incubation and prior measurements, the samples were washed 10 times with distilled water to remove background fluorescence).

We measured the fluorescence intensity of the structure as a function of the incubated biotin concentration. (FIG. S2e). The fluorescence signal saturated at 1 μM concentration after 10 min of incubation. A combined quantitative analysis via single molecule fluorescence microscopy (SMFM) and AFM provided the number of labelled molecules within the MA-SA hydrogel volume. Stepwise photobleaching was used to estimate the signal of a single ATTO655-biotin (327 ± 66 counts, FIG. S2f). AFM imaging of the grid (FIG. S2d) provides information on the volume of the fabricated structure. Under the assumption that each SA is fully functional and enables binding of four biotins and knowing the intensity of a single ATTO655-biotin, we estimate 1.83 ± 0.18 × 10^5^ streptavidins per 1.00 ± 0.05 μm³ of line volume (average over n = 10 areas). The calculation does not consider the sterical accessibility of the SA or the protein damage introduced by the methacrylation, reactivity of the photoinitiator or direct laser absorption. Unbound SA was most likely removed from the structure during the incubation and washing, therefore no significant change in the fluorescence signal of Atto655-biotin bound to the MA-SA structures was observed within 48 h. Next, the sample has been dried and AFM imaging was performed. The AFM imaging results show that, upon drying, a 3.2-fold reduction of the MA-SA line volume was measured, indicating ∼70% content of water in the line volume.

### 2.2 Application of protein-based nanostructures

Protein-based polymer structures offer great potential as scaffolds for in-vitro assays. Here we demonstrate the applicability of our polymer structures as (i) substrates for the immobilization of extracellular vesicles (EVs) and the analysis of their membrane protein composition, (ii) platform for the targeted delivery of selectively immobilized EVs to host cells via antibodies.

#### 2.2.1 Fluorescence-linked immunosorbent assay (FLISA) for the analysis of membrane proteins of EVs obtained from body-liquids

**Fig. 5:**
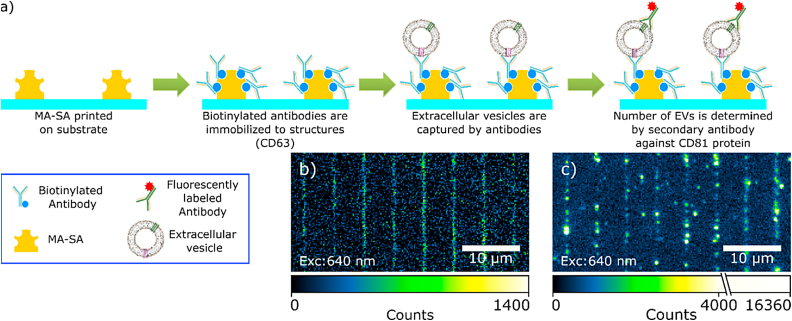
**Immobilization of EVs.** a) schematic of the immobilization procedure. MA-SA/PEG-DA lines are written on a glass substrate and incubated with a biotinylated anti-human CD63 (or CD49) antibody. EVs are captured by the antibodies. The presence of EVs is confirmed via binding of Alexa647-*anti*-human CD81 antibodies on the EVs. b) and c) shows fluorescent images before and after the incubation with the Alexa647-*anti*-human CD81 antibody.

We designed an assay for selective immobilization and screening of extracellular vesicles (EVs) based on our 2D structures after one time rewetting. The schematic procedure of these experiments is shown in Fig. 5a. First, we immobilized biotinylated antibodies on the surface of our MPL-written MA-SA/PEG-DA nanostructures, which is crucial for bioassay applications (e.g., FLISA) [57]. The structure-bound antibodies targeted transmembrane proteins in EVs (tetraspanin-30 (CD63) or the Integrin alpha 4 (CD49)). CD63 is often used as an EV marker and is present in the membrane of most EVs [[58], [59], [60]]. Contrarily, CD49 is present only in a sub-population of EVs [60]. The density of biotinylated antibodies was determined using an Alexa 647 Goat anti-Mouse secondary antibody (FIG. S3a,b). The number of immobilized antibodies per area was determined by dividing the average signal of a pixel by the signal of an individual fluorescently labelled antibody, yielding 4.1 ± 1.0 × 10^3^ antibodies/pixel (XY projection). To simulate a bodi fluid, a solution containing EVs (3 μL, 10^10^ particle per ml [61]) in 1 mg/ml of bovine serum albumin (BSA) was added to the structures and incubated for 40 min. After a washing step, the presence of the EVs on the structures was confirmed using fluorescently labelled (Alexa 647) antibodies targeting CD81 (tetraspanin-28), another known EV marker (with similar quantity per EV as CD63) [[58], [59], [60]].

Ratiometrics of CD49^−^and CD63-positive EVs revealed a 1:2 ratio of EV_CD49_:EV_CD63_, respectively. This means that the amount of EVs positive for CD81 and CD49 is 2× smaller than the amount of EVs positive for CD81 and CD63. Nevertheless, it has to be taken into account that although CD63/81 are frequently used as EV-markers, not all EVs necessarily carry both [58,59]. Nonspecific binding of the A647-antiCD81 to the structures (without presence of EVs) was not observed (FIG. S3). Our single molecule FLISA-analysis of scaffold bound EVs underscores the applicability of our approach to analyze their membrane protein composition.

#### 2.2.2 Streptavidin structures for the analysis of defined cellular EV uptake

**Fig. 6:**
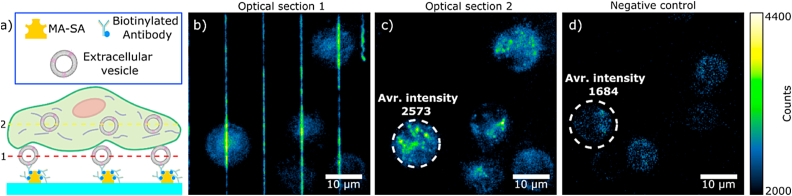
**Streptavidin structures for cellular EV uptake** a) Schematic representation of the experiment. b) Fluorescence image of eGFP-EVs immobilized on top of MA-SA/PEG-DA lines (optical section along dashed line 1 in a)). c) Fluorescent image of eGFP-EVs taken up by the HeLa cells. (optical section along dashed line 2 in a)). d) Fluorescent image of HeLa cells on a line-free area (negative control). Color scale is the same for b), c), and d) images. White dashed circles in c) and d) show individual cells and average fluorescence intensity in the respective area. The higher background fluorescence (in the cells) in panel c) is due to out-of-focus GFP signal contributions.

Next, we demonstrate that our selectively EV-loaded protein structures can be used to study the uptake of EVs in a single cell. In this experiment, 2D nanostructures were incubated with biotinylated anti-CD63 antibodies capturing fluorescently labelled EVs. The EVs carried a labelled N-terminus of the tetraspanin CD63 protein with a green fluorescent protein (eGFP-CD63 EVs, 2.8 eGFPs per EV), as described in Ref. [61]. Fig. 6b depicts the MA-SA/PEG-DA structures with eGFP-CD63 EVs (glass surface has been passivated with PEG-PLL) after an incubation with HeLa cells for 3 h at 37 °C.

We observed that part of the structure-bound eGFP-CD63-EVs were internalized resulting in the observation of an increased concentration of individual eGFP signals inside the cells (Fig. 6c). As expected, HeLa cells added to a sample without the MA-SA/PEG-DA structures do not show any fluorescent signal inside the cells (Fig. 6d). This illustrates the applicability of our polymer structures to set up a platform for uptake studies of individual EVs at the single cell level.

Additional experiments demonstrated that the 2D grids are suitable for cultivation (5 days) of HeLa cells and fluorescent readout at the single-molecule level. The supplementary FIG. S4 shows localization microscopy images of HeLa cells on top of MA-SA/PEG-DA based nanostructured lines after five days of cultivation. In these experiments, riboflavin has been exchanged with LAP as photoinitiator to reduce autofluorescence in the green emission channel, enabling single-emitter detection. For 2D localization microscopy imaging, HeLa cells were fixed, and their actin cytoskeletons were double-stained with Alexa 488- and Alexa 647 Plus-phalloidin. Thereby, a single Alexa 488 emitter exhibited a SNR of ∼19 with a position accuracy of 34 ± 9 nm, a single Alexa 647 emitter showed a SNR of ∼25 with a position accuracy of 30 ± 9 nm. Herein we prove the applicability of our new resins for 3D tissue scaffolds suitable for single molecule localization microscopy analysis.

## 3 Conclusion

We developed new resin formulations based on methacrylate BSA and streptavidin with PEG-DA and MA-HA as a cross-linker and riboflavin (vitamin B2) as a photoinitiator (or optionally LAP) and characterized their 2D/3D MPL writing capability, individual feature sizes, bio-functionality, and mechanical properties. 2D and 3D MPL structuring directly in water allowed for writing of sub-micron feature sizes. The overall larger dimensions of MA-SA based nanostructures (under wet condition) could be attributed to the larger content of water (due to high hydrophilicity) within the structures. This was indirectly confirmed by a larger decrease of MA-SA based nanostructure volume (compared to MA-BSA) after drying. The MA-SA based nanostructures showed intrinsic functionality of the streptavidin after the methacrylation and MPL fabrication. The Young's modulus of single lines was determined as a function of writing peak intensity and environmental conditions (wet and dry). The measured Young's moduli were in the kPa range and independent from writing peak intensity. We further investigated the writing threshold for different polymeric compositions, including methacrylated and non-methacrylated proteins. We show that the writing threshold is lowest for methacrylate modified polymers. In combination with the independence of Youngs modulus on writing intensity these results indicate that mostly methacrylate groups participate in the polymerization process. We showed that composition of the resin (MA-BSA/MA-SA and PEG-DA/MA-HA) allows for tuning the Young's moduli from 40 to 100 kPa for wet conditions, and from 0.15 to 1.76 GPa for dry conditions. This is similar to the range of elasticities determined for muscles (tens of kPa) up to bone ECM (hundreds of kPa) [62,63]. The combination of functional proteins and proteoglycans makes these materials suitable for mimicking the natural extracellular matrices, for tissue engineering or mechanosensitive materials. Furthermore, the fast dynamics of wetting and drying with time constants in the minute range could be interesting for various applications such as the time-dependent release of nanostructures bound species. Finally, we showed the possible application of the presented resin formulations in an FLISA assay and an uptake assay of EVs by HeLa cells. Thereby, single-molecule fluorescence microscopy was used to characterize the transmembrane protein composition of the EVs and to image the controlled delivery of the EVs to the recipient cells.

## 4 Materials and methods

### 4.1 Materials

Ultrapure and ultralow fatty acid content BSA was purchased from Nova Biologics. Formlabs standard clear resin (FLGPCL04) was purchased from Formlabs. Streptavidin (SA) was purchased from Iba (IBA GmbH, Germany), Methacrylic anhydride (94%), poly-(ethylene glycol) diacrylate (Mn = 575), Riboflavin (B2), Lithium phenyl-2,4,6-trimethylbenzoylphosphinate (LAP), 5% Picrylsulfonic acid solution and 2,4,6-trinitrobenzenesulfonate (TNBS) were purchased from Sigma-Aldrich; Biotin Atto655 (ATTO-TEC GmbH, Germany), Biotin anti-human CD63 Antibody (BioLegend, USA), Biotin anti-human CD49 antibody - CD49e (Integrin alpha 5) Monoclonal Antibody (ThermoFisher, Austria), Mouse anti Human CD81 Antibody (Bio-Rad, Austria), Goat anti-Mouse IgG (H + L) Highly Cross-Adsorbed Secondary Antibody, Alexa Fluor Plus 647 were purchased ThermoFisher, Austria. Poly(l-Lysine) Poly(Ethylene Glycol) (PLL(20)-*g*[3.5]- PEG(2)) was purchased from SuSoS AG, Switzerland.

### 4.2 Methacrylation of BSA/SA

The process for the methacrylation of proteins (BSA or SA) was adapted from previously established methods [16]: BSA (20 g, 0.3 mmol) and NaHCO_3_/Na_2_CO_3_ buffer (200 mL, 0.25 M, pH 9.0) were added to a 1000 mL round-bottom flask with a magnetic stir bar. The mixture was stirred in an ice bath in the dark until the BSA dissolved completely. Then, methacrylic anhydride (4 mL, 27 mmol) was added dropwise to the BSA solution. The reaction mixture was stirred in an ice bath for an additonal 90 min. The crude product was diluted and dialyzed against deionized (DI) water for 48 h. After lyophilization, the product was isolated as a white powder (17.8 g, 89 % yield). The procedure for methacrylation of SA was the same as for BSA with the NaHCO_3_/Na_2_CO_3_ buffer at lower pH (200 mL, 0.05 M, pH 8.2) and a final yield of 87% (17.4 g) after final lyophilization. Finally, the efficiency of methacrylation was measured by an TNBS assay (give comparable to NMR results in the estimation of degree of modification [64]) yielding 33% and 14% for MA-BSA and MA-SA respectively.

### 4.3 Protein-based resin preparation

All resins were prepared in 5 mL amber vials (Roth, Germany) to prevent auto-polymerization. The weight percentages were based on the total composition of the resin, including the aqueous solvent. As a characteristic example, we describe the preparation of the resin with 30 wt% MA-BSA and 5 wt% poly(ethylene glycol) diacrylate (PEG-DA, Mn 575). First, 1.8 g of MA-BSA was added to 3.68 mL of DI water and dissolved for 8 h at 4 °C. Then 0.3 g of PEG-DA, 0.25 wt% of Riboflavin (Vitamin B_2_) or LAP dissolved in 100 μL of DI water, and 0.05 wt% of 4-Methoxyphenol in 100 μL of DI water were sequentially added and gently stirred until dissolved (typically 20 min). The final resin formulation was covered in aluminum foil and stored at 4 °C until use.

### 4.4 Multiphoton lithography (MPL)

A customized multiphoton lithography instrument of Workshop of Photonics (WOP, Lithuania) was used for fabrication of all structures. The instrument was equipped with a fs-pulsed laser (CARBIDE, 515 nm, 1 MHz repetition rate, 290 fs pulse duration, Light Conversion, Lithuania). The specified/stated intensities are peak intensities determined in the focal plane within a diffraction-limited excitation volume, calculated from the average power measured in front of the objective lens (taking the transmission of the objective lens into account). MPL of protein-based structuring was performed in a sandwich configuration (the photoresist was kept in a chamber between two glass slide) using an oil immersion objective lens (63×, NA = 1.2 Zeiss). A 3-axis stage (AEROTECH Nanopositioner, USA) was used for sample motion. For development, the samples were rinsed with DI water to remove any uncured resin.

### 4.5 Scanning electron microscopy

SEM images were obtained using a Zeiss 1540XB SEM after evaporating approx. 10 nm of gold. In order to determine the axial structure sizes of three-dimensional structures, SEM imaging was performed either at 0° or by tilting the samples by 60°.

### 4.6 Confocal Fluorescence Microscopy

Confocal images were acquired using an inverted laser scanning microscope (Zeiss LSM 510) with a water-immersion objective lens (Plan-Apochromat, 40×, NA = 1.2, Zeiss). For recording of 3D stacks, 488 nm or 633 nm laser light was directed on to the samples via a dichroic beam splitter (488/561/633). The signals were detected by means of an avalanche photodiode using a band-pass filter (BP 505–610 nm) or a long-pass filter (LP 650 nm), respectively. Images were analyzed with Zeiss ZEN V3.6 software and with ImageJ V1.53q [65].

### 4.7 Single molecule fluorescence microscopy (SMFM)

Images were acquired using a modified Zeiss AxioObserver inverted epifluorescence microscope with an oil-immersion objective lens (63×/NA = 1.42, Zeiss). The sample was positioned on a XYZ piezo stage (P-733.3DD, Physical Instruments) with nanometer precision on top of a mechanical stage with a range of 1 × 1 cm. The sample was illuminated in the green channel with a 488 nm laser (Toptica Photonics, Graefelfing, Germany) and in the red channel with a 640 nm laser (Toptica, Photonics, Graefelfing, Germany) in wide-field configuration. The signal was detected using an Andor iXonEM+ 897 (back-illuminated) EMCCD camera (16 μm pixel size). The following filter sets were used: dichroic filter (ZT405/488/561/640rpc, Chroma, Olching, Germany), emission filter: BP525/50 or HQ 700/75 M (both Chroma Technology GmbH, Germany) for the green and red channels respectively. The parameters for dSTORM imaging were as follows: t_ill_ = 20 ms, t_delay_ = 20 ms, N_accrued frames_ = 20 000, I_640nm_ = 2.21 kW/cm^2^, I_488nm_ = 4.86 kW/cm^2^. OxEA buffer was used. The 3D localization analysis of EVs was performed using a custom-built software – 3D STORM tools [66].

### 4.8 Atomic force microscopy (AFM)

The Young's modulus measurements were performed with an atomic force microscope (JPK Nano Wizard 4, Germany) using the QI™ mode. The AFM was mounted on top of the Zeiss AxioObserver inverted epifluorescence microscope. The Young's modulus was measured with a PPP-FMR cantilever (Nanosensors, Germany; nominal spring constant = 2.8 N/m). Before each measurement, the exact cantilever spring constant and the sensitivity were determined on the substrate adjacent to the polymer lines using the contact-based calibration method (JPK, Germany).

### 4.9 Substrate preparation

All structures were written on 170 μm thick glass substrates which were plasma-treated in the presence of oxygen (for 5 min) and argon (for 10 min). The samples which were used for fluorescence measurements were additionally treated with 100 μg/mL PLL (Poly l-lysine) - PEG (ethylene glycol) (Susos, Switzerland) in PBS for 1 h for surface passivation (to minimize unspecific binding of incubated proteins). After incubation, the samples were thoroughly washed with PBS.

### 4.10 Cell culture

HeLa cells were cultured in Dulbecco's Modified Eeagle's Medium (DMEM, Thermo Fisher, USA), high glucose, supplemented with 10 % Fetal Bovine Serum (FBS, Thermo Fisher) and 1 % penicillin/streptomycin (complete growth medium DMEM-C) at 37 °C in a 5 % CO_2_ atmosphere. Prior the cell seeding, manufactured scaffolds were sterilized with UV light. HeLa cells were seeded on the structures in silicone isolator chambers (Grace Bio-Labs, USA) at a cell density of 10 000 cells in 20 μl volume. Cells were resuspended and seeded in low fluorescence FluoroBrite® medium (Gibco, USA) supplemented with 1 % EV-depleted FBS to reduce autofluorescence. Seeded structures were washed with the same medium 3× before imaging.

The samples used for Single Molecule Fluorescence Microscopy (SMFM) were washed with DMEM-C 3× prior to seeding of cells. Then 5000 cells were seeded in 20 μL of DMEM-C. Cells were washed after 4 h and 24 h and fixed immediately afterwards. Labelling was performed as follows: 3 washing steps with Phosphate Buffered Saline (PBS) (preheated 37 °C), fixation for 20 min with 4 % PFA (preheated 37 °C), 3 washing steps with PBS, permeabilization for 10 min with 0.4 % TritonX100 in PBS, 3 washing steps with PBS, blocking for 10 min with 1 % Albumin from Chicken egg (ACE) in PBS, incubated for 20 min with 66 nM Phalloidin Alexa 647 Plus in ACE, washed thoroughly with PBS, incubated for 20 min with 66 nM Phalloidin Alexa 488 in ACE, washed thoroughly with PBS.

### 4.11 Extracellular vesicle preparation

The EVs were prepared as previously published [61]. HEK293T cells (ATCC #CRL-3216) were cultured in DMEM high glucose, supplemented with 10 % FBS and 1 % penicillin/streptomycin (complete growth medium DMEM-C) at 37 °C in a 5 % CO_2_ atmosphere. Transfection of HEK293T with the GFP-CD63 plasmid (Addgene #62964) for over-expression of the GFP-labelled EV marker CD63 was performed with 1 μg of plasmid DNA and 2 μL Endofectin Max reagent (Genecopoeia, USA) in 6-well plates according to the manufacturer's instruction. For generation of a stable cell clone, the cells were selected by adding 1 μg/ml puromycin. After expansion of the stably transfected cells for several weeks, cells were sorted by fluorescence-activated cell sorting (FACS) for eGFP + cells. Single cell derived clones were isolated and characterized by flow cytometry and microscopy. For EV isolation, parental or transfected HEK293T cells were cultured to a sub-confluent state in DMEM-C. FBS was centrifuged at 110 000×*g* for 12 h to deplete the medium of EVs from bovine origin (EV-depleted FBS). For collection of the conditioned media, the media was changed to OptiMEM containing 2 % EV-depleted FCS, and supernatants of ∼130–150 x 10^6^ cells were collected after 24 h. The number of cells and the viability of EV producing cells was verified with a CASY cell counter (OMNI Life Science, Germany). Cellular debris, apoptotic bodies and larger EVs were removed from the supernatants by centrifugation at 200×*g* (5 min), 2000×*g* (10 min) and 10 000×*g* (30 min) at 4 °C. Small EVs were obtained by ultra-centrifugation at 110 000×*g* (70 min) at 4 °C (Sorvall MX150, Thermo Fisher Scientific, USA) in a fixed angle rotor (Hitachi S58A-0095, Chiyoda, Japan). The resultant pellet was resuspended in PBS, and concentrated by ultracentrifugation, and subsequently resuspended in 30 μL of PBS. Samples were stored at −80 °C.

## Supporting information

SEM imaging of 3D suspended lines; details on streptavidin functionality testing – comparison of fluorescence before and after incubation of fluorescently labelled biotin; image and analysis of the single biotin intensities; fluorescence of the nanostructures as a function on the biotin concentration. Details of the methods for detecting methacrylation efficiency of proteins. dSTORM images of actin in HeLa cells grown on top of the MA-SA nanostructures.

## Funding

This work was funded by the Austria Science Fund (10.13039/501100002428FWF) project P 31827.

## CRediT authorship contribution statement

**Dmitry Sivun:** Conceptualization, Data curation, Formal analysis, Methodology, Supervision, Visualization, Writing – original draft, Writing – review & editing. **Eljesa Murtezi:** Conceptualization, Data curation, Investigation, Methodology, Writing – review & editing. **Tina Karimian:** Data curation. **Kurt Hurab:** Data curation. **Maryam Marefat:** Data curation. **Elena Klimareva:** Data curation, Formal analysis. **Christoph Naderer:** Data curation, Formal analysis. **Boris Buchroithner:** Conceptualization, Data curation. **Thomas A. Klar:** Conceptualization, Supervision, Writing – review & editing. **Georgii Gvindzhiliia:** Data curation. **Andreas Horner:** Data curation, Formal analysis, Writing – review & editing. **Jaroslaw Jacak:** Conceptualization, Funding acquisition, Project administration, Supervision, Writing – original draft, Writing – review & editing.

## Declaration of competing interest

The authors declare that they have no known competing financial interests or personal relationships that could have appeared to influence the work reported in this paper.

## Data Availability

Data will be made available on request.

## Appendix A Supplementary data

Multimedia component 1 Multimedia component 1

Movie S1 pulling individual MA-BSA line with AFM cantilever. (AVI). Movie S1
