# Supplementary material for: Multiphoton lithography with protein photoresists
Source: Mater Today Bio. 2024 Feb 10;25:100994. doi: 10.1016/j.mtbio.2024.100994 (PMC10879783; doi:10.1016/j.mtbio.2024.100994)
Supplement: Multimedia component 1 [file mmc1.docx]

Supporting Information

3D multiphoton lithography with protein photoresists

Dmitry Sivun ^a*^, Eljesa Murtezi ^a^, Tina Karimian ^a^, Kurt Hurab ^a^, Maryam Marefat ^a^, Elena Klimareva ^a^, Christoph Naderer ^a^, Boris Buchroithner ^a^, Thomas A. Klar ^b^, Georgii Gvindzhiliia ^b^, Andreas Horner ^c^, Jaroslaw Jacak ^a^


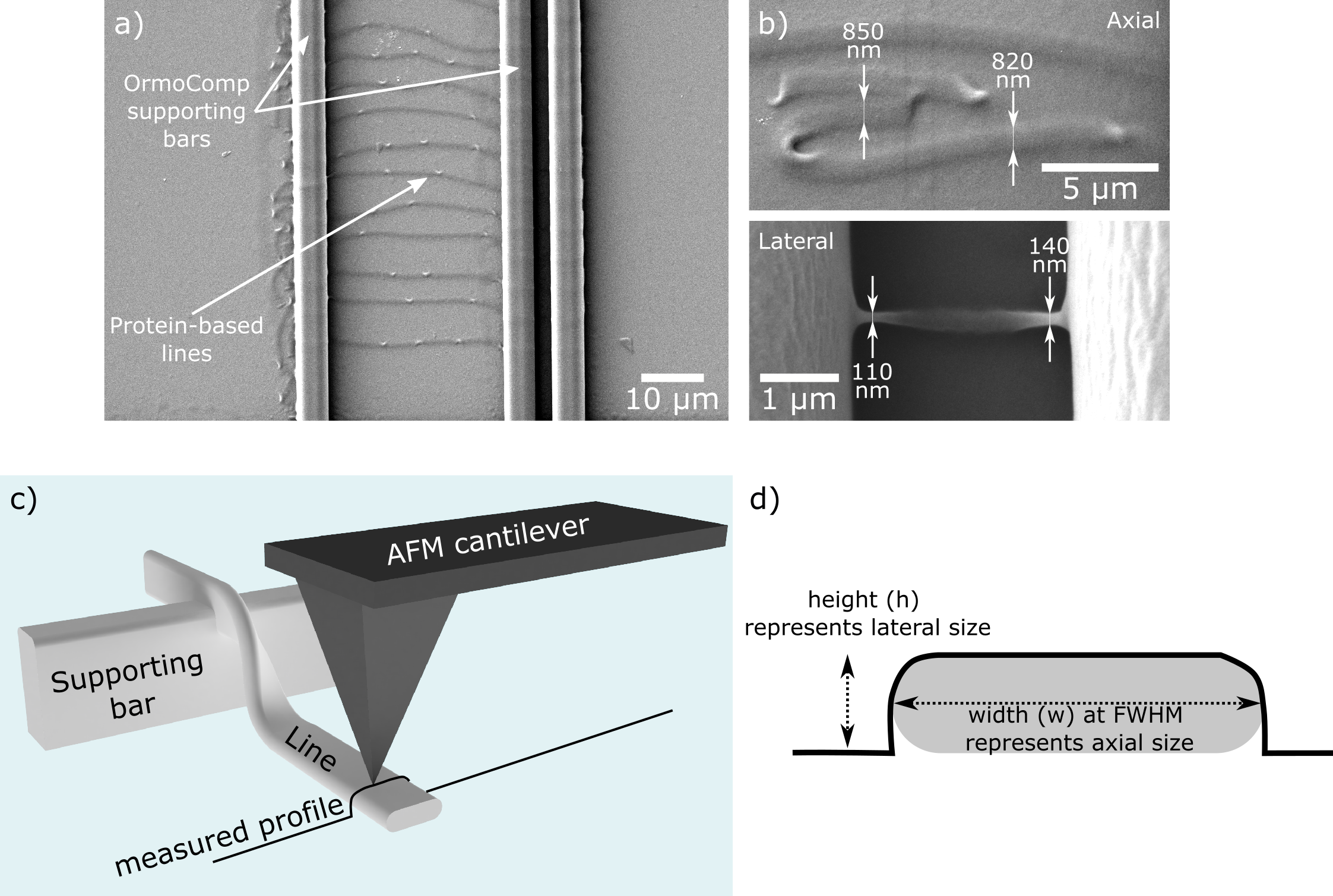


**FIG. S1.** a) SEM image of 15 3D hanging protein-based lines between OrmoComp supporting bars. b) SEM images of MA-BSA lines showing lateral and axial dimensions, respectively. c) Schematic illustration of the of arrangement protein-based lines after development (line is tilted by 90° and lay down on glass substrate) and AFM measurement configuration. d) Schematic drawing of the line (gray ellipse) and measured AFM line profile (black line)

**Table S1.** Mean dimensions of smallest achievable lines in wet and dry states. SD: standard deviation.

| MA-BSA-based lines | | | | |
| --- | --- | --- | --- | --- |
|  | Mean Lateral, nm | SD Lateral, nm | Mean Axial, nm | SD Axial, nm |
| Wet | 205 | 26 | 1042 | 92 |
| Dry | 104 | 18 | 920 | 169 |
|  |  |  |  |  |
| MA-SA-based lines | | | | |
|  | Mean Lateral, nm | SD Lateral, nm | Mean Axial, nm | SD Axial, nm |
| Wet | 349 | 14 | 2313 | 230 |
| Dry | 92 | 5 | 1907 | 110 |


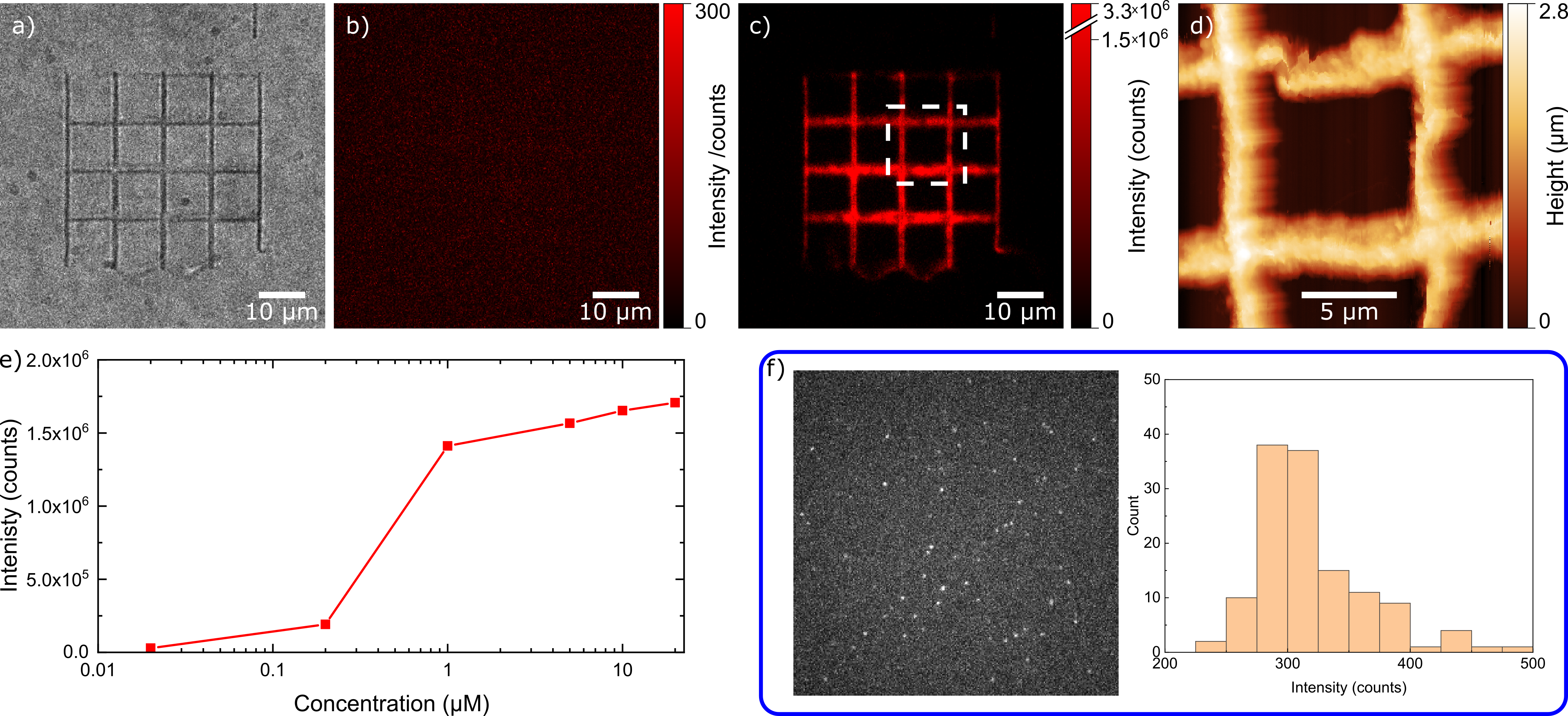


**FIG. S2.** **MA-SA Functionality**. a) shows a bright field image of the MA-SA 2D grid. b) and c) shows the corresponding fluorescence image of the same grid, before and after incubation with fluorescently labeled (ATTO 655) biotin, respectively. d) shows an AFM image of the grid unit cell (marked with a white dashed line in c). e) shows the intensity of the MA-SA lines as a function of the fluorescently labelled (ATTO 655) biotin concentration used for incubation. f) shows a fluorescence image (left panel) of single ATTO655 biotin fluorophores on a glass substrate and the resulting intensity histogram; average single emitter intensity: 327±66 counts.

**
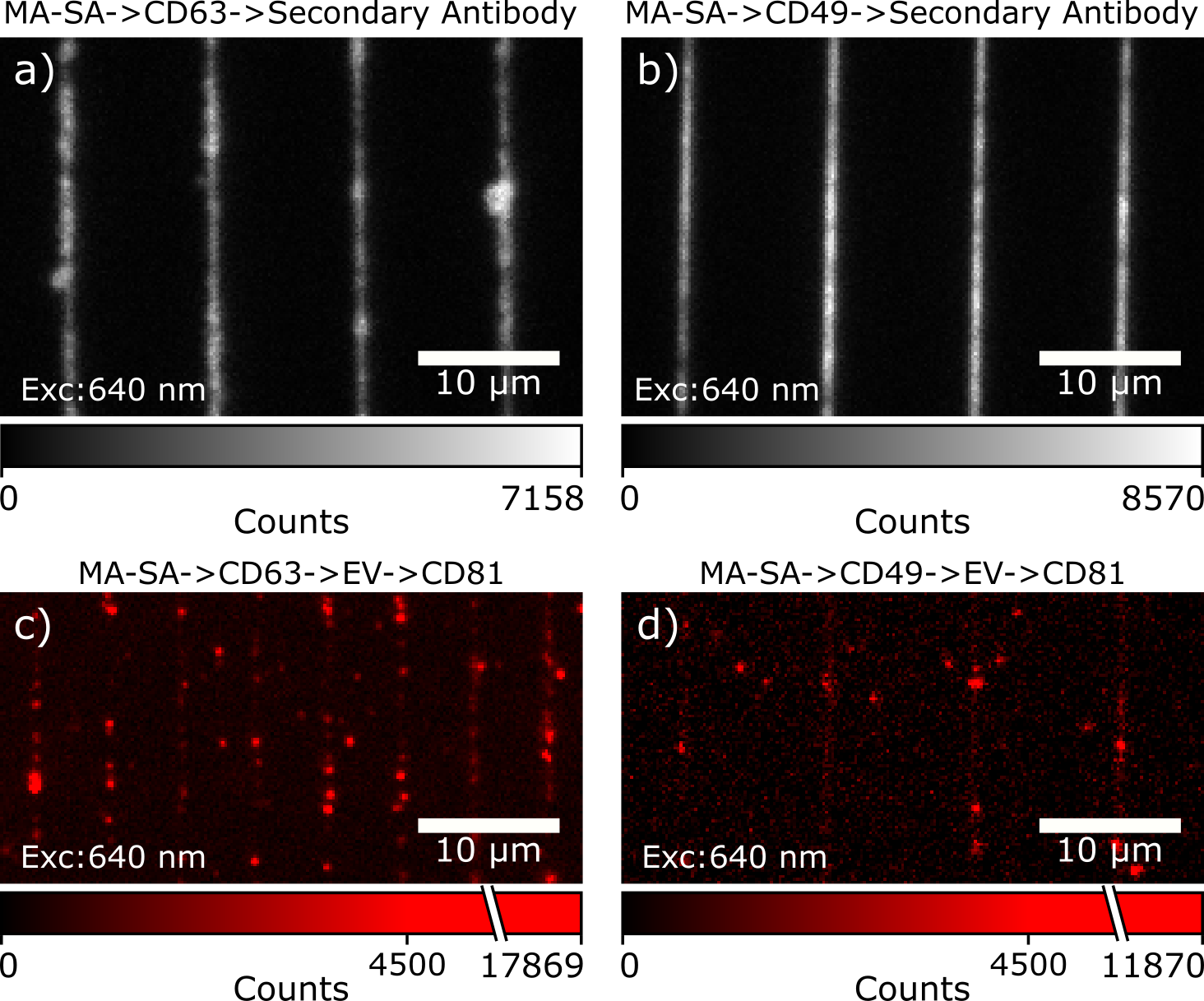
**

**FIG. S3.** The density of biotinylated anti-CD63 (a) and anti-CD49 (b) antibodies bound to MA-SA lines is determined by incubating the lines with Alexa 647 Goat anti-Mouse secondary antibody. c) and d): fluorescence images of CD63-EV-CD81 and CD49-EV-CD81 FLISA constructs, respectively. Both images show the sparse cd81/cd63 and cd49/cd63 positive subpopulations of EVs.

**
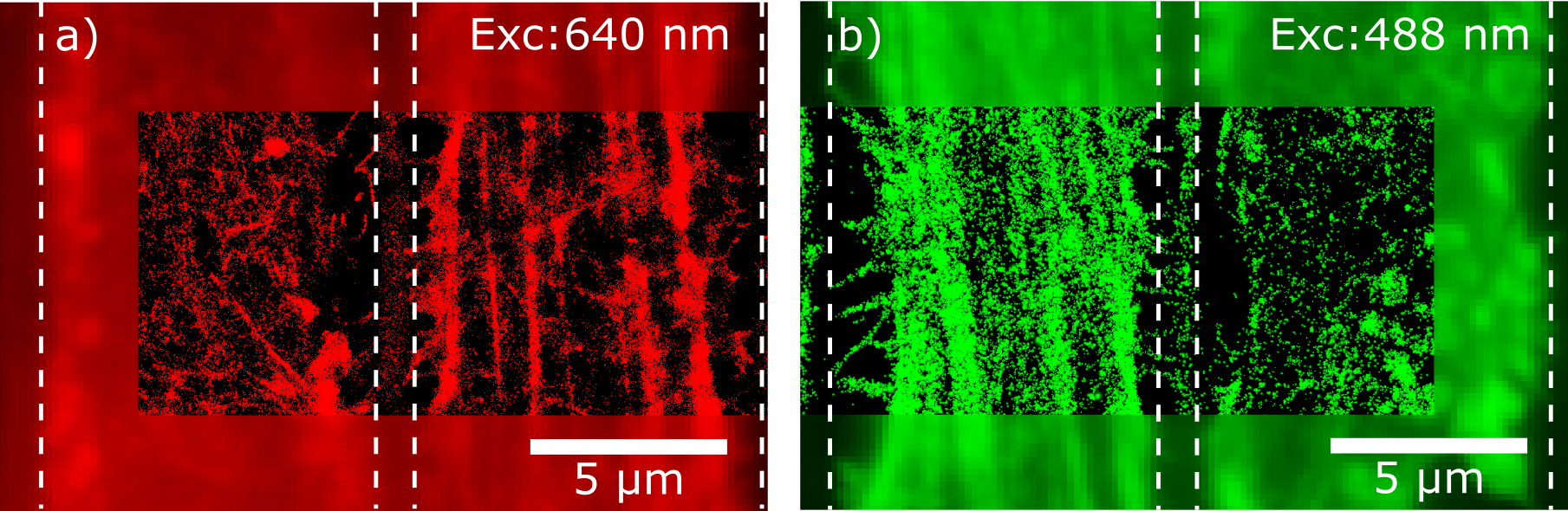
**

**FIG. S4.** Overlap of standard fluorescence images with single molecule localization images of HeLa cells on top of MA-SA/PEG-DA nanostructured lines (cultivated for 5 days). HeLa cells were fixed, and actin was double stained with phalloidin a) Alexa 647 Plus and b) Alexa 488. The zooms of both color channels are not from the same corresponding cell areas. The SNR was 25 and 19 for the red and blue channel, respectively. The lateral position accuracy was 30 ± 9 nm and 34 ± 9 nm for the red and blue channel, respectively. White dashed lines show positions of the MA-SA lines.

**Calculation of percent functionalization of the available lysines of BSA and SA**

The TNBS assay is a method used to quantify primary amino groups by N-trinitrophenylation of primary amines, which have high absorption at 335 nm. Primary amines react with TNBS to form a compound that absorbs strongly at 335 nm. BSA, MA-BSA, SA and MA-SA were dissolved in carbonate-bicarbonate buffer at a concentration of 20 μg/mL. A 0.01% (w/v) solution of TNBS (0.25 mL) was added to 0.5 mL of each protein solution. The samples were incubated at 37 °C for 2 h. To quench the reaction, 0.25 mL of 10% SDS and 0.125 mL of 1 N HCl were added to each sample. The absorption of each solution was measured at 335 nm with a UV−vis spectrophotometer. The absorbance of the MA-BSA and MA-SA was compared to the absorbance of native BSA and SA accordingly, and the percent functionalization was then calculated using the equation below.

$$\%functionalization=\frac{\mathrm{Abs}_{BSA/SA}- \mathrm{Abs}_{{BSA}_{MA}/{SA}_{MA}}}{\mathrm{Abs}_{BSA/SA}} x 100\%$$
